# Supplementary material for: Patterns of Intron Gain and Loss in Fungi
Source: PLoS Biol. 2004 Nov 30;2(12):e422. doi: 10.1371/journal.pbio.0020422 (PMC532390; doi:10.1371/journal.pbio.0020422)
Supplement: Table S1 — Also available at http://genes.mit.edu/NielsenEtAl/. (4.3 MB ZIP). [file pbio.0020422.st001.zip › NielsenEtAl/html/78.html]

AN5455.1.NCU00336.1.MG04995.1.FG07262.1


```
 CLUSTAL W (1.82) Multiple Sequence Alignments - Introns Inserted


Sequence 1: NCU00336.1	1788 aa
Sequence 2: MG04995.1	1796 aa
Sequence 3: FG07262.1	1806 aa
Sequence 4: AN5455.1	1801 aa
Alignment Length: 1833 aa
Number Identitical Residues: 465 aa
Alignment Score (without introns) 30209


MG04995.1 	MATSLAAQLAQIAAKSRSSLNVKAQKAAHTKSLIFEPRIAAGQSYQTIYTACRDGFEELC
NCU00336.1	-MSSLASQLAQIAANSRSTLNTKVLKAAHSKSLIFEPRVAATQTYPEIYSICLEGFEELC
FG07262.1 	MATSLAAQLAQVAANSKSTLNVKAQKAAHSKSLIWEPRVAATQSYQNLYTTCIQGFEELC
AN5455.1  	MASSLAAQLSQIAANSTNQLNLKAQRLAHSKSLIFDKRVAGSQDFDTIYDICYDGFRELC
          	  :***:**:*:**:* . ** *. : **:****:: *:*. * :  :*  * :**.***

MG04995.1 	QLDARFTPFSNTLFSEQSQDEDRSQMTQAENKELDRRVDSFLRLVGSRLRLMPAIKAIEW
NCU00336.1	NLDSRFTKFTQSLWSPQSQEADRTQMSAAENAALDKHVEAFLHLCGSRLRLMPTIKAIEW
FG07262.1 	QLDARFAPFHSTIFSEESQNQDRTQLTAAENEELDRRVEAFLRLAGSRLRLMPAIKSIEW
AN5455.1  	QLDFRFAQFERSIFSEQSKVQDRTEMNVEQNRELDSVLEAFLALVGGRLLLSPAVKAVEW
          	:** **: *  :::* :*:  **:::.  :*  **  :::** * *.** * *::*::**

MG04995.1 	LIRRFR2IHEFNTSTLLATFLPYHSIPAFVTLLSILPADLPQQYKFLAPYVRSLTAPPRA
NCU00336.1	LIRRFR~VHEFNTAALIATFLPYHTIPAFVTLLSILPANIPKEYRFLDPYIRSLTAPPRA
FG07262.1 	LIRRFR2IHEENTQALLLTFLPYHSIPAFATLLSILPNKIPHDFRFLDPYIRSVTSPPRS
AN5455.1  	LIRRFR2VHEYNTRFTILTFLPYYSTPVFLNLLAILPDDLPSALKVLIPYKRSAINPVRQ
          	****** :** **   : *****:: *.* .**:*** .:*   :.* ** **   * * 

MG04995.1 	AIVYQATHHREFLTAITEYTLDACRNKQQYPALVSFWAGVMTEAVNGRIDINRSGRQGVQ
NCU00336.1	AIVQQATNRPELLTAISQYTLDSCKYQMEYPGLISFWGGVMVEATNGLLDKYRSGRRSIQ
FG07262.1 	VLVRQAIMHPVFLTLISEYTLQSCRMQYNYPTLISFWAGIMTEAVGGILDKTRSGRANVQ
AN5455.1  	ALVQNAISNRDLITTLNNYVLQVCRQRAHHHALLAFWAGIITEGVAGMLDSSRSGRRNVE
          	.:* :*  .  ::* :.:*.*: *: : .:  *::**.*::.*.. * :*  **** .::

MG04995.1 	LDNDLALIALVSKPLGDAMTMKDVPDIQIAAYMVVSLLAAKANFDDKLLSVFMEQLVIGW
NCU00336.1	IENDNALMQQLGPVLSDAMVMKSSPGLQIASYMVVTILAAKGGLADNALTAFMDQLVHGW
FG07262.1 	SENDQNLLHRLGPIFAESLVMKKVPSIQVASYMAIAVFVAKGNLEDNAVTAFMDQTVYGW
AN5455.1  	KQKHDDIILQILPVLNDGFAMKDVSELVIGCYMVCVVLAQKAELQDRVLDGLMEAVTGSW
          	 ::.  ::  :   : :.:.**. . : :..**.  ::. *. : *. :  :*:  . .*

MG04995.1 	TAETVRPGLVCLSILAQYRAPKQLSSRVAKALLKMDDLPQRLVDASKQCRVDKLAYALVL
NCU00336.1	TPETMRPGLVTLCIISQHRSAKQLSARVTKALLKVPEVASVMNEIGKDHRVDKLANGLAL
FG07262.1 	TTETVRPALVVLATLAQYRSAKQMSGKTTKALMKVTDIGNLLVEIGQDRRVDKLANGLCL
AN5455.1  	TEETMSSGLICVAVLAQQKPDPILPKRVFKAMLRMKDPLKQLADISTEYKTSQLLLGLVA
          	* **: ..*: :. ::* :.   :. :. **:::: :  . : : . : :..:*  .*  

MG04995.1 	ALADRLAKKGDARGLPIIKSILLLELLQEKLGKVAFKSLLRAALKMTDEVDQDGSARKQL
NCU00336.1	ALVDRLHKKGDVRSLPVVNSLLLGNVLREKQIKVVYKSLLVAAHRINDQVDQDGAIRKEL
FG07262.1 	ALIERLTKKGDARGLATIITILSSSVLKDKQIAVIFKSLILTAHRLNDDNDEDGALRQEL
AN5455.1  	GCVDSLSTQKDSARLELLSSMFKSQLLNDADTAKAMTFVLQAASAVGGTISLD--TQTQI
          	.  : * .: *   *  : :::  .:*.:       . :: :*  : .  . *   : ::

MG04995.1 	GSTLVLLSQSAGATGVMVRQVIEDEDYDIEGLELRLDTSFGPRRLLEDKPAEEDAKDLEV
NCU00336.1	GTVLVSLSQAGGEVGDIVRATIDEVDFDIDALELTLGASIRPKLAVEDAPEAAAEDNNTP
FG07262.1 	GSALIALSQNEGKSGDTIQSVIEEVKFDIEELEMKLDLSFRSRRLPESKNDQDMSNGIEE
AN5455.1  	AEIVQHFSRSS-SLQPVFQKVIDESSIDLSVIEYNLQTVIES-VPVNKAIEDVEMEDADK
          	.  :  :*:        .: .*:: . *:. :*  *   : .    :.       ..   

MG04995.1 	RQDPKETLEAAIERLAVPPRSVKSFLSQKSDQLFDDMCKVLLSVATDPDD----LLKFDE
NCU00336.1	KVDKEAQVAQNFEKLSKLKPQTASCFAEEPLDLLEELYSLFLSVAANESN----LQKFDE
FG07262.1 	RKTP-QDLSLLLKELSGRSESLSPCLVPQPSEIFDEFSHIFFTVVSESSQNTELLSEFEL
AN5455.1  	TETATDGYDSALESLAKESSFSTSFLTAQSIPVFDKLVQTFALSAGSPER----VSAFVE
          	           :: *:       . :  :.  :::.:   :   . . .     :  *  

MG04995.1 	APFLCRQTAHDDSYYFSFYMRVWCGPYPTLIRSAALEMAKRRLKQGDCAALDLQAMIPYC
NCU00336.1	APVLSRPQAPTKLFYASFYMRLWCGSLPTLAKVAALDRVKNLLKDEEFATLDFQAVVPYA
FG07262.1 	NPKLHRQAAFKDCTYFSFFIRIWTGPYPALARVAALDMVKKRLKAGDAGKTDLQALLPYC
AN5455.1  	LPVLCKANATTSPQFLSFFVRVFTGMYPAGVKAAALKTISSIVSSAAWSDVDVQALLPFM
          	 * * :  *  .  : **::*:: *  *:  : ***.  .  :.    .  *.**::*: 

MG04995.1 	VAALSDPAKKVRQAAADLITVLSKSNAEGIETAKRPIWASKQLYEQSNEQTWMNPAASRA
NCU00336.1	IVALSDPAKKVRRAAAELVTVLG-SFYETKPSKARRVWGSEGLYAKNAAVNWLDFDATKS
FG07262.1 	ISALSDPSKRVRKSAADLIAVLTGLYIPPVPAKGLEIWGERGLYGKSKEFASLASDVVAK
AN5455.1  	LIALADPSERVRSGAVDALANIGKVVDK---KKKSGVWARDSLYGKSMHIPWLSSSDFQK
          	: **:**:::** .*.: :: :              :*.   ** :.     :       

MG04995.1 	LLQEIIVPALEESVLDEEHISNVLRSHLQSTK------DSATG-PQLSSAVRLSIFSCIT
NCU00336.1	LIHSVLIPSLEESILHEDHILAALTNALESSK------SKDGDKKHLSHSTRFAIFKFLS
FG07262.1 	VLHLQILPTVEECVMDPEHITAVLRAAIEQGKYQSKPDSSLDKKDHLSQPGRLSFLQFLA
AN5455.1  	ILERAVLPELEECRSDGEHIGRALENALRGAA--------SDSASAIKKPLRLAFFTYLC
          	::.  ::* :**.  . :**  .*   :.                 :. . *::::  : 

MG04995.1 	SHVVHTPLLAVKSRLLKPTNQVDSVSKATRTSLLLPLLRWWASLSSTEAAQLCERERIDE
NCU00336.1	SHVVGTPLIAVKLRLLQSLNQIKSISGTSRTDLLLSLLQWWARLSEAEAQQLLAREAVDE
FG07262.1 	SHVVHTPLILVKSRLLKAVNEIRGVSTTTRTQLLLPALQWWASLNEDEATKLCSSERLEK
AN5455.1  	SHAVHLPLFAPRAGLLNLLNRVDKAGGTTRTKELEPLLKKWRDMSEQEVAEVHEKEQISV
          	**.*  **:  :  **:  *.:   . ::**. * . *: *  :.. *. ::   * :. 

MG04995.1 	SKFNQSCVNVAVANDKPGLETLFEIITNSSAWTREGLVEAAFARTRKIWSTIKSDSKLKL
NCU00336.1	ASVNNAFVDVVIANNEAGLRLIFELIRDSNVITRNGLVQSLFSRVQKIWSSMKAETQFST
FG07262.1 	SDMDNCLVDIVVANDVAGLEFFMEFLREPEHTEKAGLIRAIFARMRKIWSSMKPDVKFTA
AN5455.1  	SDFEAQVLKTVTPKEKDSINLLLSTVTPYSPSLRASFVSSVFNRISEIWGKVPEDRQITA
          	:..:   :. . .::  .:. ::. :   .   : .:: : * *  :**..:  : ::. 

MG04995.1 	ALSMLDLAQTPATAGSSAEFVSEEAADFLRNVDLTTNILNAFLESLQDIPKITTDSPATK
NCU00336.1	ARALLTLSQAVHPTSSEPDVIATEATDVLHKVELTTDILLDFLESLYDDIKKATEKPATK
FG07262.1 	AEQLLEISQDSAVSADSKSIVSTEAADFLRNVRLTTDILDYFLQSIPTGTKMITEPPANK
AN5455.1  	AEKLFELS-----TQASESPLVDNARDLLRRVELPGPVLLNYLQQIPASITDIDSLGPAP
          	*  :: ::     :  . . :  :* *.*:.* *.  :*  :*:.:    .   .  .  

MG04995.1 	RRRTSSSDHSRGVSLQATNAVKAALNKVTFVLQLVEGSKPAEHPELLQSLFVTLADLQVL
NCU00336.1	RRRVGSSEKS--VDSQSPADVSASLSKATFVLELVQESEPAKHPELLPSLFTTLSELQHL
FG07262.1 	RRRISSSGIDRGLVTQVTPEISQELRKLTFILQLVENSDPAEHPDLLNGLFTALSELQHF
AN5455.1  	KRRRTSQNNMVAMTTKDEAKLSKLMDKMTFILELVDGSSPEAHPELTEWLFQTLAALHHF
          	:**  *.    .:  :    :.  : * **:*:**: *.*  **:*   ** :*: *: :

MG04995.1 	RSQVGSELGYLQNLVLSSLLAMMPTYKNNKSLKIDTSVGHGDVLVQCIQKSSSPAVQNSA
NCU00336.1	RTVVGSELGYLQSLVLSSLLAMMPAYKDSKELTIDPAAGHGDILASCIQRSSSPTVINAA
FG07262.1 	GTVVGSELGYLQNLILRSLLAMMPVYKSNKKLKIHSSSSYGDLLVRCIQKSSSPVVQNAA
AN5455.1  	KSQIQSGMSYLLSLTLGSLLAIVNRSRASSKPQFDTSVVRADLVVDCVRTTESPQVQNTA
          	 : : * :.** .* * ****::   : ...  :..:   .*::. *:: :.** * *:*

MG04995.1 	LLLVASLANTAPDVVLHSVMPIFTFMGGSVLRQSDDYSAHVVVQTIKEVIPPLVETFRKS
NCU00336.1	LLLVASLARTAPDVVLHSVMPIFTFMGSSVLKQADDYSAHVVNQTVKEVIPPLIETFRKS
FG07262.1 	LLLVASLAAAAPNVVLHSVMPIFTFMGVSVLRQSDDYSAHVVSQTIKEVIPPLIQSMRQG
AN5455.1  	LLLVAGLSVIAPELVLHSVMPIFTFMGSSVLRKDDDYSVSVIDQTIDQVVPALIQSLRHQ
          	*****.*:  **::************* ***:: ****. *: **:.:*:*.*::::*: 

MG04995.1 	RRNLVTSAAELLSSFVIAYEHIPSHRKRDMFISLIENLGPKDFLAALVAMFVDKYGTTDS
NCU00336.1	RRNLVASTAELLTSFVVAYEHIPSHRKQDLFITLIENLGPEDFLFAVLAMFVDKYGATDN
FG07262.1 	QKNPLAGACDILVSFTTAYEHMPPHRRQGLFVALVETLGPEDFFHAIIGMLVDKYGSSDA
AN5455.1  	KRDVVSGTSELLLSFTAAFEHIPSHRRLRLFHALITKLGTEEFLFAVLAMLANRYSMDKA
          	::: ::.:.::* **. *:**:*.**:  :* :*: .**.::*: *::.*:.::*.  . 

MG04995.1 	MVAFCKDLMNSFGVEVQLQSLVMLLDLVSDCLQPKPTLAATLF--NRN-TDDDQDLHKTA
NCU00336.1	MLAFTTQIIGSFSVEIQLQTLIKHLDLISDIFKPKPVLSAALL--AKVDSNSEQDVVKLA
FG07262.1 	LLQFVIELLNHFSIATQLQTLVKLLDLIADLFKAKPGISFVLLGISENGDDKDKDLQEIA
AN5455.1  	VLVLMTGLVSDADATVELSTYSKFLNLVGDSLKSKPGISQVLLG---IGSDDGREPHKVA
          	:: :   ::.  .   :*.:    *:*:.* ::.** :: .*:.    . :. ::  : *

MG04995.1 	LKELTLLPKVLSSRQLRTEVSQLAGRDDMEASKFRELYASLLEKILTLAETVKTNK-ALH
NCU00336.1	TKQLTLLPKLLVNRRLRHEISGLAEKDDMESVKIRELYAQLLEGVLTLAGTVKPKKDTLY
FG07262.1 	LKQLSAFPVFLANKRLRSQIGGLMENDDMEASRLRELYATLLENILVLADTVKADT-TLH
AN5455.1  	ADLLRALAYLFKHSSLKVKIARALTTETDDSERIRALFSNILEQVLAIGESMQSVK-PVH
          	 . *  :. .:    *: ::.     :  :: ::* *:: :** :*.:. :::. . .::

MG04995.1 	ARCSDTLAGLLNLLSIGEFIKAVENLLDRPSISLRQKVLRTLEVRVDQESNTDADSRTVL
NCU00336.1	TRCAEALSNLLNLLSIAEFIKSVEALLDRPNVILRQKVLRALERRVDSESINNPKSREAL
FG07262.1 	GRCGQALHNHLNLLSIGEFIKAVENLLDRPDMGLRQKVLRALEVRVEQESNTDPASRAVL
AN5455.1  	QAAGDVLSGLFSTLTTIDFLDTIEALLKRPDDALRRKVLSLLATRLQQSPERDGASQTRM
          	  ..:.* . :. *:  :*:.::* **.**.  **:***  *  *::...  :  *:  :

MG04995.1 	LAFLPQLTAVIRDSDDIAYKHTAVACVDKIAEKYGKKDLEAVAAAATTIAGDCCLGQPDK
NCU00336.1	LAFLPQLTAVIRESDDMNYKHTAVNCVDKIAEKYGKKDLDAVAAAAATIAGDSCLGQPSQ
FG07262.1 	LAFLPQLTAGIRESTDVRYKHTAVTCVDKISEKYGKKDIEAVVAAATTIAGEHCLGQPEQ
AN5455.1  	LDFLTVLVDIVQSSPDILLKHAAVTCIDRITEKYGKKEPSMVTSAAQVVASASCIGQEDD
          	* **. *.  ::.* *:  **:** *:*:*:******: . *.:** .:*.  *:** ..

MG04995.1 	RLRVMALLCLASLVDVLQDGIVPTLPVSLPKALSYLSESLQGEK-EPELHNAVYSFFESL
NCU00336.1	ELRVMALLCLASLVDVLQDAIVPVLPIAIPKALGCLEESIKAEKPDGALHNAAYAFMAAL
FG07262.1 	RLRVMALLCLTSLVDVLQDAIVPVLPSALPKAVKYLDESLEDDDRDEELHVASYGFISAL
AN5455.1  	RIRINGVLCLASMVEVLGQAMIPALPEVLNRSLALLELSLETNKVNARLHDAVFTLFSAL
          	.:*: .:***:*:*:** :.::*.**  : :::  *. *:: :. :  ** * : :: :*

MG04995.1 	AVHVPYMLTKTYIGQLLAVSNVSAEANMSDESSQARFGCLQLLANQVDANTMLAALEQNW
NCU00336.1	AQHIPYMISGAYLDRLLVCSNASAAAGLNEECRDSRTDCLQFVAKLIEGKVLFTALEKNW
FG07262.1 	AQYLPYMLS-TYLDRVLEVSNKSAEIDLEDNTRESRVDCLHFVAKQLEAKEIFSALDRNW
AN5455.1  	FVHLPYMVSASHLDRLLVLSFKSAASDE-DLDNENRQEALHFMARKVDMAVALASIERNW
          	  ::***:: :::.::*  *  **  .  :   : *  .*:::*. ::    ::::::**

MG04995.1 	AQAVNAGFS0AVEEYLKLLATVLDKHPSTIIAKHISTLSTIFLSALDLRRNVQSKDTVSI
NCU00336.1	ANAASSGYL0ALEEYLHVLGTALDKHPKSSIAKNTTLFTGIFLNAFDLRR------SGVL
FG07262.1 	ESAKSAGFS0AVAEFLDVLGLAIDKHPKSSISKNAMLLSSIITKVFDLRRQERVKGEFGE
AN5455.1  	TQAVSAGPS~ATHEVLDAISLSIEKHPKSATMKNLSVLTTILFRAFDLRR-EQTQSSESA
          	 .* .:*   *  * *. :.  ::***.:   *:   :: *:  .:****    ..    

MG04995.1 	AALAKLTEIEALINDVALKMIYKLNDSKFRTVFTQLMEWVATGLPKDDKLGKVLRHQSVY
NCU00336.1	SSTQELEKIELLINETSLKMIYKLNDAAFRPMFSHLMEWSTTGLPKSDLAGKAQRQVSTY
FG07262.1 	QDLLRLSALDASANDKALKMIYKLNDAAFRPVFVQIIEWSNSGLPKSDRLGRSLRQFSVY
AN5455.1  	FDASDLEEIEDLINDVTIKMIYKLNDTAFRPIFIKLVEWAT-GLPEKNTQGGLARLTTFY
          	     *  ::   *: ::********: **.:* :::**   ***:.:  *   *  : *

MG04995.1 	SFLLAFFDNLK~DVVASYASYIIDDATAILKASDPSKMEDRELWQLVLKTLARCFEHDSG
NCU00336.1	GFLQHFFENLK~SIVTSYASYIIDSAVKILSAPLTSDDVLKTLRSRVLRTLTKCFEHDQD
FG07262.1 	GFLEAFFGTLK~SIVTNYATYIVEDAVKILTTVDLKNPEERQLWTRVLQTLGNCFEHDQD
AN5455.1  	RFLQVFFGTLQ0SIVTGYASYIIESVVSVLETASPSNPNTKSLWLATMRMLRSAFEHDQD
          	 **  ** .*: .:*:.**:**::... :* :   ..   : *   .:: *  .****..

MG04995.1 	G~FWQVPAHFEAVSGLLVEQLEHAAA--LGELPGGDLVQNAVVGLAEAAASRDHRKELNA
NCU00336.1	G~FWQAPAHFNAVAPVLVAQFGHAAG--ADNCTN-DLVL-AVVELAAAADSKEHHKEINS
FG07262.1 	D~FWQAPAHFGAVGPVLMGQFTHAG---MVEVTD-DLIP-TTVELASAADSQAHQKELNS
AN5455.1  	E1FWQSPSHITKISTPLISHLRHATSTTTGALVATETIP-TITELAVAADSTDNHKELNT
          	  *** *:*:  :.  *: :: ** .::       : :  : . ** ** *  ::**:*:

MG04995.1 	AVLRRLRSPSASV-------------------RLAAVRCEQSLTDTLGEDWLEMLSEMLP
NCU00336.1	ALLKHLRSEQAAV-------------------RLAVIKCEQELTARLGEEWLQSLPEMLP
FG07262.1 	ALLKHLRSESAAV-------------------RLAAVKCQQALTDRLGEEWLSMLHEMLP
AN5455.1  	VLMRFLRPSSASLSSSSTKRAGPGLGGDNPQTRIAALKTEQALTEHLGEDWLALLPEMLP
          	.::: **. .*:::::::. :... ......:*:*.:: :* **  ***:**  * ****

MG04995.1 	YISELQDDDDEDVEKETHRWITKIEAILGESLDAMLQ
NCU00336.1	FISELQDDDDEVVERENRRWIVGIEETLGESLDNMLQ
FG07262.1 	RISELQEDDDEVVERETHRWIVMIEEVLGESLDAMLQ
AN5455.1  	YISELMEDEDEGVEREVRKWVKQIEGVLGERLDDMLT
          	 **** :*:** **:* ::*:  **  *** ** **
```
